# Dynamics in the Strawberry Rhizosphere Microbiome in Response to Biochar and *Botrytis cinerea* Leaf Infection

**DOI:** 10.3389/fmicb.2016.02062

**Published:** 2016-12-22

**Authors:** Caroline De Tender, Annelies Haegeman, Bart Vandecasteele, Lieven Clement, Pieter Cremelie, Peter Dawyndt, Martine Maes, Jane Debode

**Affiliations:** ^1^Plant Sciences Unit, Crop Protection, Institute of Agricultural and Fisheries ResearchMerelbeke, Belgium; ^2^Department of Applied Mathematics Computer Sciences and Statistics, Ghent UniversityGhent, Belgium; ^3^Plant Sciences Unit, Crop Husbandry and Environment, Institute of Agricultural and Fisheries ResearchMerelbeke, Belgium; ^4^Bioinformatics Institute Ghent From Nucleotides to Networks, Ghent UniversityGhent, Belgium

**Keywords:** biochar, rhizosphere, plant growth, above-ground infection, bacteria, fungi

## Abstract

Adding biochar, the solid coproduct of biofuel production, to peat can enhance strawberry growth, and disease resistance against the airborne fungal pathogen *Botrytis cinerea*. Additionally, biochar can induce shifts in the strawberry rhizosphere microbiome. However, the moment that this biochar-mediated shift occurs in the rhizosphere is not known. Further, the effect of an above-ground infection on the strawberry rhizosphere microbiome is unknown. In the present study we established two experiments in which strawberry transplants (cv. Elsanta) were planted either in peat or in peat amended with 3% biochar. First, we established a time course experiment to measure the effect of biochar on the rhizosphere bacterial and fungal communities over time. In a second experiment, we inoculated the strawberry leaves with *B. cinerea*, and studied the impact of the infection on the rhizosphere bacterial community. The fungal rhizosphere community was stabilized after 1 week, except for the upcoming Auriculariales, whereas the bacterial community shifted till 6 weeks. An effect of the addition of biochar to the peat on the rhizosphere microbiome was solely measured for the bacterial community from week 6 of plant growth onwards. When scoring the plant development, biochar addition was associated with enhanced root formation, fruit production, and postharvest resistance of the fruits against *B. cinerea*. We hypothesize that the bacterial rhizosphere microbiome, but also biochar-mediated changes in chemical substrate composition could be involved in these events. Infection of the strawberry leaves with *B. cinerea* induced shifts in the bacterial rhizosphere community, with an increased bacterial richness. This disease-induced effect was not observed in the rhizospheres of the *B. cinerea*-infected plants grown in the biochar-amended peat. The results show that an above-ground infection has its effect on the strawberry rhizosphere microbiome, changing the bacterial interactions in the root-substrate interface. This infection effect on the bacterial rhizosphere microbiome seems to be comparable to, but less pronounced than the effect of biochar-addition to the peat. The biological meaning of these observations needs further research, but this study indicates that biochar and an above-ground pathogen attack help the plant to recruit rhizosphere microbes that may aid them in their plant growth and health.

## Introduction

Biochar is the solid coproduct of a biomass pyrolysis process for the production of biofuel (Gravel et al., 2013). Biochar was found to act as a fertilizer in container-grown plants, i.e., as a source of nutrients for plant growth (Altland and Locke, 2013a; Locke et al., 2013), and interacted with the fertigation solution by retaining or releasing nutrients in a nutrient-specific way (Altland and Locke, 2012, 2013b). Studies show that adding biochar to growth substrate can not only enhance crop productivity and performance (De Tender et al., 2016), but can also reduce crop susceptibility to diseases (Meller Harel et al., 2012; De Tender et al., 2016).

Biochar addition has also been proven to affect the soil microbial community structure (Kolb et al., 2009; Anderson et al., 2011; Lehmann et al., 2011; Abujabhah et al., 2016). The effect of biochar addition on the microbial community structure and diversity of the rhizosphere (the environment immediately surrounding the plant roots) is less well-understood, however. The rhizosphere plays an important role in the growth, nutrition, and health of plants due to the action of microorganisms which closely interact with the plant root (Philippot et al., 2013). Furthermore, it has been proposed that adding biochar to peat or soil not only enhances plant growth through a direct effect by nutrient addition, but also by an indirect effect on the root-associated bacteria, which shift in composition after biochar addition (De Tender et al., 2016; Egamberdieva et al., 2016).

In Belgium and The Netherlands, strawberry cultivation relies mainly on cold-stored strawberry plants used as planting material. They are taken from a nursery field (field soil) in December/January and cold-stored at −1.5°C. Starting in January until the end of August, strawberry plants can be planted and cultivated, which greatly extends the traditional production season and provides an important economic benefit (Lieten et al., 1995; Lieten, 2013). The cold-stored strawberry plantlets are generally planted into peat substrate. The main cultivar used in this cultivation system is “Elsanta” (Lieten, 2013). This cultivar is susceptible to fungal diseases, including *Botrytis cinerea* (gray mold), which is one of the most destructive diseases on strawberry worldwide. Controlling the disease with fungicides is difficult, mainly because of the long latency period between inoculation and the appearance of symptoms, the prolonged and overlapping flowering and fruiting periods, the explosive fungal development that occurs at or near harvest time and the onset of fungal strains resistant to fungicides (Sutton, 1990).

In a previous study, strawberry plantlets of the cultivar Elsanta were grown for 13 weeks in peat with and without biochar. Adding biochar to the peat resulted in a lower susceptibility of the plants toward *B. cinerea*. After these 13 weeks an increased bacterial biodiversity was noted in the strawberry rhizosphere and a shift toward bacterial genera including species previously reported to be involved in biological control and induced resistances. In addition, there were also small differences in the chemical composition of the peat vs. the biochar-amended peat (De Tender et al., 2016).

However, these biological and physicochemical changes were only measured at the end of the experiment (13 weeks after planting). The moment that the shift in bacterial community composition took place within the rhizosphere remained unknown. Furthermore, the effect of the *B. cinerea* inoculation on the rhizosphere community has not been monitored, as only rhizosphere samples of non-inoculated plants have been studied. Therefore, two major questions still remain: (1) what is the dynamics of the biochar-mediated shift in the strawberry rhizosphere microbiome, and (2) is there an effect of an above-ground infection with *B. cinerea* on the rhizosphere microbiome of the strawberry plants? An additional aim was to gain more insight in the role of fungal communities in the strawberry rhizosphere.

To study the rhizosphere microbiome, both bacterial and fungal communities were studied using 16S rDNA V3-V4 and ITS2 metabarcoding, respectively. Two experiments were set-up: (1) a time course experiment in which the effect of biochar and the dynamics over time on the rhizosphere microbiome were studied, and (2) an inoculation experiment in which the effect of *B. cinerea* leaf inoculation on the bacterial community in the rhizosphere was studied. For both experiments, plants were grown in peat or biochar-amended peat for 13 weeks, in which the rhizosphere was sampled at the end in the inoculation experiment, and at eight pre-set time points during the plant growth cycle in the time course experiment. In addition to the rhizosphere microbiome analysis, chemical properties of peat and biochar-amended peat were analyzed and the effect of biochar on plant and root growth, fruit yield, and disease susceptibility against *B. cinerea* inoculation on both plant leaves and fruits were analyzed.

## Materials and methods

### Chemical characterization of biochar, peat, and amended peat

Biochar prepared from holm oak at 650°C for 12–18 h was kindly provided by Proininso S.A. (Malaga, Spain). This biochar consists of 72.4% dry matter (DM; %/fresh), 77.8% organic matter (%/DM), and 74.2% C (%/DM) and was previously used and fully characterized by Huang et al. (2015), De Tender et al. (2016), and Vandecasteele et al. (2016). Peat used in the strawberry assays was NOVOBALT white peat 100% (AVEVE Lammens, Wetteren, Belgium).

The substrate was sampled at different time points (described below) during the strawberry experiments. Dry matter content was determined according to EN 13040. Electrical Conductivity (EC; EN 13038) and pH-H_2_O (EN 13037) were measured in a 1:5 soil to water (v/v) suspension. Determination of organic matter content and ash was done according to EN 13039. Extraction (1:5 v/v) of water soluble nutrients and elements (NO_3_-N, NH_4_-N, Cl, SO_4_, and PO_4_-P) was done according to EN 13652, and measured with a Dionex DX-600 IC ion chromatography (Dionex, Sunnyvale, CA), and for NH_4_-N with a Skalar San++ mineral N analyzer.

### Strawberry experiments

Peat was used as growing medium and used as either pure growing medium (298 g peat) or mixed with 3% biochar (9.4 g biochar + 289 g peat). Additionally, 1.33 g L^−1^ fertilizer (PGMix, Peltracom, Ghent, Belgium) and 3 g L^−1^ lime (Dolokal extra, Ankerpoort NV, Maastricht, The Netherlands) were added to both the peat and the peat/3% biochar mixture. No additional fertilizer was applied during plant growth. Both substrates were wetted to obtain 40% water-filled pore space (WFPS), and bulk density was adjusted to 200 g L^−1^. Each mixture was put in a closed bag and pre-incubated at 15°C for 1 week. Subsequently, 1.5 L pots were filled with the mixed substrates and a cold-stored bare-root strawberry (*Fragaria* × *ananassa*, cultivar Elsanta) transplant was planted in each pot. The plants were then arranged in the greenhouse in a completely randomized design and grown at 20°C for up to 13 weeks. Every week, the moisture content of the substrate was adjusted to 40% WFPS based on mass loss.

Two experiments were done. In a first experiment, referred to as the “time course experiment,” in total 24 plants were grown in peat and 24 in peat amended with 3% biochar. Plants were sampled in a completely randomized way for rhizosphere microbiome analysis at nine time points: before planting and 1, 2, 3, 6, 9, 10, 12, and 13 weeks after planting. Three replicates were sampled for each time point × growing medium combination. After 9 and 12 weeks of plant growth, the *B. cinerea* bio-assay was done on the plant leaves as described below.

In a second experiment, further referred to as the “the inoculation experiment,” 24 plants were cultivated in peat and 24 in peat amended with 3% biochar (*n* = 48). Half of the plants in each treatment were inoculated with *B. cinerea* on a leaf at 12 weeks after planting using the method described in the next paragraph. This resulted in four treatments: peat non-inoculated (peat NI), peat inoculated (peat I), peat amended with biochar non-inoculated (Peat+BC NI), and peat amended with biochar inoculated (Peat+BC I). Additionally, fruits were harvested, weighed and inoculated with *B. cinerea* as described below. At 1 week after inoculation (i.e., after 13 weeks of plant growth) the rhizosphere of six biological replicates per treatment were sampled (see “Sampling and DNA extraction” below).

At 13 weeks after planting, the strawberry plants were collected and weighed [fresh weight (FW) and dry weight (DW, 48 h at 70°C)] and the root development was measured. This was done by observing the root systems that show up at the substrate surface when removing the pot. Depending on the number of visible lateral roots (lateral roots and root hairs), a 0–3 developmental score was given, with 0, no lateral roots; 1, a few lateral roots; 2, lateral roots all over the substrate surface; and 3, substrate surface fully covered with lateral roots.

### *Botrytis cinerea* bio-assay

Plants and strawberry fruits were inoculated with *B. cinerea* at pre-set time points for both experiments. Plant leaves were inoculated using the method of Meller Harel et al. (2012). Briefly, the *B. cinerea* isolate 895 (Debode et al., 2013) was cultured on Potato Dextrose Agar (PDA) at 20°C for 4 days. Agar discs (4 mm) containing pathogen mycelium and conidiophores were cut out from the colony edge and placed, mycelium side down, on the surface of three young fully expanded strawberry leaves per plant, with one disc per leaflet. Control leaves were inoculated with sterile PDA plugs. All plants were sprayed with water and each pot (volume: 1.5 L) was covered with a plastic box for 1 week to create conditions of high humidity. The resulting lesions on the leaflets were recorded 1 week after inoculation using a 0–4 disease scale with 0, 0% of the leaf area infected (no symptoms); 1, <25% of the leaf area is affected; 2, 25–50% of the leaf area is affected; 3, 51–75% of the leaf area is affected; 4, >75% of the leaf area is affected (Supplementary Figure 1). After scoring the infection, inoculated leaves were removed from the plant.

Inoculation of the strawberry fruits was based on the method of Bhaskara Reddy et al. (2000). Briefly, individual ripe strawberry fruits were inoculated with 20 μl conidial suspension (2 × 10^5^ conidia mL^−1^) of *B. cinerea* and incubated at 11°C under humid conditions. When the first symptoms appeared, the strawberries were evaluated daily and spoiled fruits were discarded to avoid secondary infection. The area under the disease progress curve (AUDPC) was calculated for the infected fruits (Campbell and Madden, 1990).

### Sampling and DNA extraction

In the time course experiment, the rhizosphere was sampled from strawberry roots before planting, followed by sampling at 1, 2, 3, 6, 9, 10, 12, and 13 weeks after planting. Three replicates were taken at each time point for the plants grown in peat and biochar-amended peat. For the inoculation experiment, the rhizosphere of strawberry plants was sampled after 13 weeks of plant growth. For each condition (peat NI, peat I, peat+BC NI, and peat+BC I; in which NI, non-inoculated; I, inoculated; BC, biochar addition) six biological replicates were taken. Rhizosphere sampling was done according to Lundberg et al. (2012), in which 25 mL of root material was used. The resulting pellets (250 mg), which are considered as the rhizosphere sample, were immediately used for DNA extraction with the PowerSoil DNA isolation kit (Mo Bio, Carlsbad, USA), according to the manufacturer's instructions. DNA was stored at −20°C until further use.

### 16S rDNA and ITS2 amplicon sequencing

Amplicon sequencing of the bacterial and fungal rhizosphere populations was done on the V3–V4 fragment of the 16S rRNA gene and the ITS2 gene fragment, respectively, using Illumina technology (Illumina, San Diego, CA, USA). Using an amplification and dual-index PCR successively, fragments were amplified, and extended with Illumina specific adaptors, which is described in detail in De Tender et al. (2016) and Debode et al. (2016). Each PCR step was followed by a PCR product clean-up using the CleanPCR reagent kit (MAGBIO, Gaithersburg, MD, USA).

Final libraries were quality controlled using the Qiaxcel Advanced, with the Qiaxcel DNA High Resolution kit (QIAGEN, Germantown, MD, USA), and concentrations were measured using the Quantus double-stranded DNA assay (Promega, Madison, WI, USA). The final barcoded libraries of each sample were diluted to 10 nM and pooled in a 2:1 ratio for bacterial and fungal libraries respectively. Resulting libraries were sequenced using Illumina MiSeq v3 technology (2 × 300 bp) by Macrogen, South-Korea, using 30% PhiX DNA as spike-in.

### Sequence reads processing

Demultiplexing of the amplicon dataset and removal of the barcodes was performed by the sequencing provider. The raw sequence data is available in the NCBI Sequence Read Archive under the accession number SRA399532 for the time course experiment and SRA416875 for the inoculation experiment. A detailed description of the sequence read processing can be found in De Tender et al. (2016). Briefly, Trimmomatic v0.32 was used for removing the primers (Bolger et al., 2014). Raw Illumina forward and reverse reads were merged using the program PEAR (Zhang et al., 2014). To extract the ITS2 sequences from the complete amplicon sequence, which includes parts of the neighboring, highly conserved, ribosomal genes, the ITSx program was used (Bengtsson-Palme et al., 2013). In the following steps, different programs of the Usearch software v7.0.1090 were used (Edgar, 2014). Merged sequences were quality filtered. Next, sequences of all samples that needed to be compared to each other were merged, dereplicated, and sorted by size. Clustering the reads into Operational Taxonomic Units (OTUs) was done using Uparse, with an identity level of 97% for bacterial sequences and 98.5% for fungal sequences (Edgar, 2014). Chimeras were removed from the V3–V4 fragments using Uchime with the RDP Gold database as a reference (Edgar et al., 2011). Finally, sequences of individual samples were mapped back to the representative OTUs using the “usearch_global” algorithm at 97% identity, and then converted into an OTU table (McDonald et al., 2012).

### Downstream data analysis and statistics

All statistical analyses were done using the R statistical software, version 3.2.2 (R core team, 2014).

Chemical substrate properties were analyzed as a two-way ANOVA with biochar treatment and time as the two factors within the time course analysis, and biochar treatment and the presence of infection as the factors in the inoculation experiment. To use the ANOVA analysis, first Levene's test was used to study homogeneity of the variances.

The plant properties data was analyzed for seven dependent variables: plant fresh weight, dry weight, root development, number of fruits picked, fruit weight per plant, leaf lesions, and AUDPC for the fruit rot. Homogeneity was tested using Levene's test. If variances were equal, a *t*-test was used, otherwise the Wilcoxon-rank sum test was used.

OTU tables of the 16S V3-V4 and ITS2 amplicon sequencing were analyzed using the QIIME software package (v1.9.0) (Caporaso et al., 2010a). Taxonomy was assigned with the script “assign_taxonomy.py” using the uclust method considering maximum three database hits, with the Silva v119 97% rep set (as provided by QIIME) as reference for the bacterial sequences and UNITE v7 (dynamic) for fungal sequences (Caporaso et al., 2010b; Quest et al., 2012; Kõljalg et al., 2013).

For the microbial analysis, both differences in community composition and in community richness were studied.

Within the time course analysis, we first focused on the total community composition differences between groups, in which treatment of biochar and time were indicated as the main factors in the experiment. The multivariate analysis was done using the specific R package vegan (version 2.0-10) (Oksanen et al., 2010). The dissimilarity matrix, based on the Bray-Curtis dissimilarity index, was calculated from the OTU table as generated by Usearch, for both the bacterial and fungal sequences. Using the betadisper function, the homogeneity of the variances was checked on this dissimilarity matrix. Further, the significance of biochar treatment, time and the interaction effect between treatment of biochar and time were analyzed using PERMANOVA analysis, in which the Bray-Curtis dissimilarity index matrix was used as input.

Secondly, we assessed differential abundance using likelihood-ratio tests. We tested for (1) the effect of time within non-biochar treated samples and the biochar treated samples, separately and (2) the effect of treatment within each time point. The analyses were done upon clustering the bacterial and fungal OTU tables generated by QIIME at family level for research question 1 above and on genus level for research question 2 above. In a filtering step OTUs with low count number in most samples were removed. For both fungal and bacterial OTU tables on family or genus level, only those families/genera with a count of four in at least three samples were kept for analysis. Normalization is based on the trimmed mean of *M*-values (TMM) in which we correct for effective library size of the count tables (Robinson and Oshlack, 2010). This normalization takes the sequencing depth into account and corrects for the presence of highly abundant families. The counts are modeled OTU by OTU using a negative binomial (NB) model with main effects for time and biochar, as well as a biochar × time interaction. The effective library size was used as an offset in the model for normalization purposes, hence, all model parameters have an interpretation in terms of changes in relative abundance. Empirical Bayes estimation of the overdispersion parameters of the NB model was adopted using the quantile-adjusted conditional maximum likelihood (qCLM) method by shrinking the OTU-level overdispersion toward the common dispersion across all OTUs. Statistical tests were adopted on the appropriate contrasts of the model parameters to assess the research questions of interest. We adopted the Benjamini-Hochberg False Discovery Rate procedure to correct for multiple testing. All of these analyses were done using edgeR package, version 3.12.0 (Robinson et al., 2010).

Third, statistical differences in richness between groups were studied for both the bacterial and fungal sequences. Rarefaction analysis was done using the “alpha_rarefaction.py” script of QIIME. A plateau was reached at 50,000 sequences for the bacterial OTUs and 20,000 sequences for the fungal OTUs. Richness of the bacteria and fungi was determined on rarefied data, for which the number of sequences was set on the reached plateau. The temporal evolution of richness is expected to be non-linear. Therefore, an additive model is used with two thin plate regression spline components:

$$\begin{array}{l} {\text{y}}_{\text{i}}={\text{f}}_{\text{a}}\left({\text{t}}_{\text{i}}\right)+{\text{x}}_{\text{i}}{\text{f}}_{\text{b}}\left({\text{t}}_{\text{i}}\right)+\varepsilon_{\text{i}}, \end{array}$$

with y_i_ the richness of observation i, t_i_ the time in weeks at which observation i is taken, f_a_(t_i_) a smoother to model the evolution in average richness, x_i_ an indicator variable, which is x_i_ = 0.5 when observation i is treated with biochar and x_i_ = −0.5 when observation i originates from the control treatment and f_b_(t_i_) a smoother modeling the average difference in richness between biochar amended medium and the control medium. The knots of the splines are placed at the nine observed time-points (t = 0, 1, 2, 3, 6, 9, 10, 12, 13 weeks) and the smoothness penalty is tuned by exploiting the link between smoothing and mixed models (Ruppert et al., 2003; Wood, 2005). Upon fitting, the additive model can be used to study the average evolution of the richness in peat [f_a_(t_i_) − 0.5 f_b_(t_i_)] and biochar amended medium [f_a_(t_i_) − 0.5 f_b_(t_i_)], separately. However, it is more appealing to assess the first derivative of these average richness profiles since it indicates if the richness is increasing or decreasing over time. Next, inference on smoother f_b_(t_i_) is adopted to study the effect of biochar addition over time. Note, that the additive model can provide inference on average richness, difference in richness and their first derivatives at any timepoint *t*, however, if we want to assess these effects at multiple time-points we have to adjust for multiple testing. We address the multiple testing issue by using a grid based approach for constructing approximate simultaneous confidence intervals (Ruppert et al., 2003) and adjusted *p*-values (Yang et al., 2013). To control the multiple testing burden, we consider a grid that is spanned by the nine observed time-points in the experiment.

Within the inoculation experiment, the total community composition differences between groups were analyzed similar to the time course experiment, using PERMANOVA analyses. Within these experiments, both the main effect of biochar treatment and infection with *B. cinerea* were studied, as the interaction effect between the factors. Second, we wanted to test which genera show differences in relative abundances between: (1) peat NI vs. peat I, (2) peat NI vs. peat + BC NI, (3) peat I vs. peat + BC I and (4) peat + BC NI vs. peat + BC I. To do so we used the bacterial OTU tables clustered on genus level, as generated by QIIME. Differential abundance at OTU-level was assessed using the EdgeR procedure described above. Third, the significant differences in richness were estimated on the rarefied data, obtained from the OTU table as generated by QIIME. Data was rarefied at 50,000 sequences. Equality of variances between groups was tested using Levene's test. Statistical differences in richness were analyzed using a linear model with main effects for infection and biochar addition and the infection × biochar interaction.

## Results

### Effect of biochar on chemical properties of the substrates

Within the time course experiment, changes in chemical parameters of the strawberry substrates (peat and peat amended with 3% biochar) were measured over time. Additionally, the effect of biochar amendment in peat was studied for each time point (Table S1). Over time, no significant changes were observed in the chemical parameters within 13 weeks of plant growth for both the peat and biochar amended peat treatment. The addition of biochar to the growing medium, however, significantly raised the pH, and reduced the amount of NH_4_-N, independent of the time point at which the parameters were measured.

In the inoculation experiment, the effects of biochar incorporation and *B. cinerea* leaf infection on peat chemical parameters were measured at the end of the experiment, after 13 weeks of plant growth (Table S2). The above-ground infection did not affect the plant available nutrients and pH of the peat. Similar to the first experiment, adding biochar to the growing medium of strawberry raised the pH. Other chemical parameters were not altered by the addition of biochar, however.

### Effect of biochar on plant growth, root growth, and strawberry production

Seven dependent plant properties were measured at the end of the strawberry growth cycle: plant fresh weight, dry weight, root development, number of fruits picked, fruit weight per plant, leaf lesions, and AUDPC for evaluating fruit rot caused by *B. cinerea*. For these results, measurements of strawberry plants of both the time course and inoculation experiment were pooled, as no interaction effect of biochar treatment and experiment was noticed.

Addition of 3% biochar to peat significantly increased the development of lateral roots, the number and weight of the strawberry fruits and the resistance of the fruits to *B. cinerea*. Biochar had no effect on leaf and petiole fresh and dry weight nor on the leaf lesions caused by *B. cinerea* (Table 1). For the leaf lesions, it should be noted that the infection rate was very low (mean score was <1, with about 15% of the leaf area was affected). The effect on disease severity after adding 3% biochar to fresh and dry weight of peat corresponds to the results of De Tender et al. (2016). Root development and number of fruits were not assessed in that study.

**Table 1:** **Properties of strawberry plants grown for 13 weeks in peat with (3%) and without biochar**.

	**Leaf and petiole weight (g per plant)**		**Root development (0–3)**	**Fruits per plant**		**Disease severity (*****B. cinerea*****)**	
	**Fresh**	**Dry**		**Amount**	**Weight (g)**	**Leaf lesions (0–4)**	**Fruits decay (AUDPC)**
Peat	42.19 ± 2.15	16.92 ± 0.73	**1.54** ± **0.18**	**2.23** ± **0.22**	**12.43** ± **1.15**	0.68 ± 0.07	**65.31** ± **6.09**
Peat + 3% biochar	44.78 ± 1.91	18.09 ± 0.80	**2.76** ± **0.15**	**4.22** ± **0.35**	**21.95** ± **1.96**	0.69 ± 0.07	**45.46** ± **2.27**
*P*-value	0.18	0.36	<**0.001**	**0.02**	**0.02**	0.90	**0.04**

*Properties that are statistically different between control peat and peat with 3% biochar treatment are indicated in bold*.

### Time course experiment

Within this experiment, we questioned how rhizosphere microbiome compositions evolve during growth of the plants, by analyzing successive time points. Subsequently, the effect of biochar mixed in the peat substrate was analyzed for each time point, comparing the rhizospheres in peat and in the peat/biochar mix. To study these effects, changes in community composition as well as differences in richness are analyzed on both the bacterial and fungal community.

#### Shifts in bacterial and fungal community structure

The bacterial and fungal community composition of the strawberry rhizosphere were studied during a 13 week growth period. The main effects of the biochar treatment and time, and their interaction effect were estimated using the complete OTU dataset. The OTUs were homoscedastic between groups, and therefore general effects could be studied using PERMANOVA analysis.

For the bacteria, the interaction effect between biochar addition and time was extremely significant (*p* < 0.001). In other words, both the time and biochar treatment affect the bacterial community composition; further, the temporal changes in community structure are different for the biochar-amended and unamended peat. For the fungi however, only a significant effect of time (*p* < 0.001) was measured; biochar treatment did not alter the fungal community composition of the rhizosphere.

#### Temporal variation in bacterial and fungal community structure

Both the bacterial and fungal communities of the rhizosphere changed significantly over time, both for the plants grown in peat and biochar-amended peat separately.

**Figure 1:**
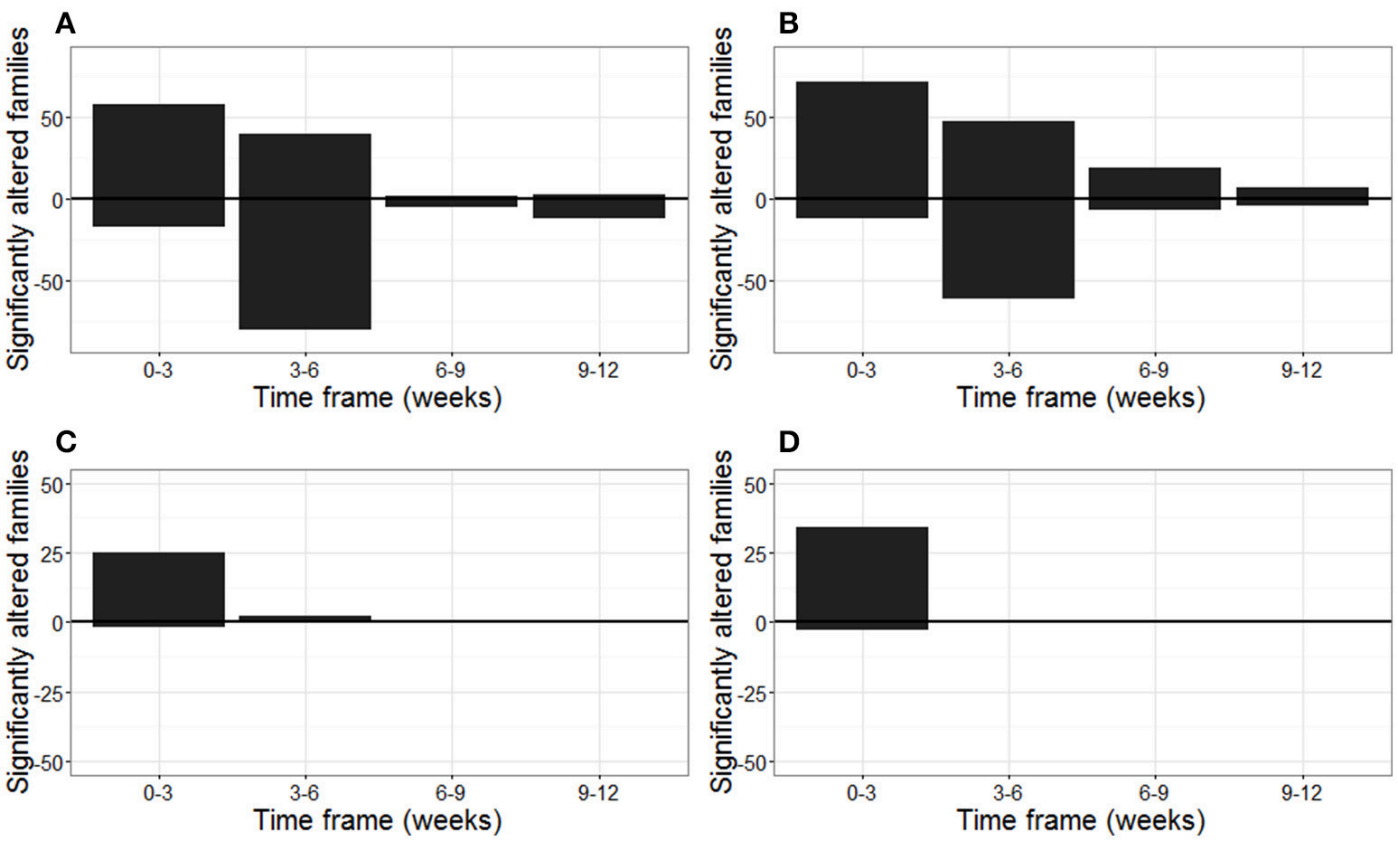
**Number of bacterial and fungal families of the strawberry rhizosphere that altered significantly over time (weeks)**. The number of families that increased and decreased in relative abundance within a three week timeframe are shown above and below the horizontal line, respectively. Number of significantly altered bacterial families in the rhizosphere of strawberry grown in **(A)** peat, and **(B)** biochar amended peat. Number of significantly altered fungal families in the rhizosphere of strawberry grown in **(C)** peat, and **(D)** biochar amended peat. Plants were leaf-inoculated with *B. cinerea* at the beginning of weeks 9 and 12.

These temporal changes in bacterial and fungal community are represented in Figure 1, in which the number of significantly altered families within a 3-week timeframe are represented. Especially within the first 6 weeks of plant growth, a high number of bacterial families changed in relative abundances over time, both for plants grown in biochar-amended or unamended peat (Figures 1A,B).

In total 77 bacterial families changed significantly in relative abundance between at least two successive time points within the strawberry rhizosphere of plants grown in peat or biochar amended peat. In total, 45 of these families represented at least 0.1% of the total community for at least one time point (Supplementary Figure 2). Within the first 3 weeks of plant growth, a reduction in the relative abundance of especially the Flavobacteriaceae, Sphingomonadaceae, and Microbacteriaceae and an increase in relative abundance of the Rhizobiales incertae sedis were noticed. Between weeks 3 and 6, a reduction in the relative abundance of Chitinophagaceae was mainly observed, especially in the non-amended peat. The period from week 6 onwards, was characterized by a significant increase in relative abundance of the Acidobacteriaceae for plants grown in peat and the peat/biochar mixture and of the Acetobacteriaceae and Rhodospirillaceae solely in the biochar-amended peat (Supplementary Figure 2).

In contrast with the bacteria, the main changes in fungal community composition occurred during the first week of plant growth, both for the plants grown in peat and in biochar-amended peat (Figures 1C,D). This was characterized by a prominent decrease of the Amphisphaeriaceae and an increase of the Morteriellaceae and Lasiosphaeriaceae (Supplementary Figure 3). From week 3 onwards, no important shifts occured in the fungal community, with the exception of Auriculariales Incertae sedis, which increased significantly between weeks 3 and 9, and then decreases again at the end of the strawberry growth period in week 13 (Supplementary Figure 3). In total, 38 fungal families of the strawberry rhizosphere altered in relative abundance between at least two successive time points for plants grown in peat or biochar amended peat. In total, 10 of these families represented at least 0.1% of the total fungal community and are represented in Supplementary Figure 2.

#### Effect of biochar amendment per sampling time point

An effect of biochar was only observed for the bacterial rhizosphere. This effect was further studied for each time point, using the OTU table clustered on genus level. The bacterial genera that changed significantly in relative abundance (*p* < 0.05) within each time point as a response to the addition of biochar are listed in Table 2. Three groups could be discriminated, representing different effects of biochar on three time frames. The first group (group A), consisted of genera that changed in relative abundance within the first 6–9 weeks of plant growth as a response to the biochar supplemented to the peat. Group B contained genera that changed in relative abundance from week 6 of plant growth untill the end of the experiment. Finally, group C contains bacterial genera that change in relative abundance from week 9 to 13 of plant growth. The major changes in the bacterial community of the rhizosphere due to the addition of biochar were observed from week 6 onwards, however (groups B and C). In general, these were mainly increases in relative abundances noticed for those genera that differed significantly between the biochar and non-biochar treatment at the specified time point. In contrast, in group A we observed decreases in relative abundance due to the addition of biochar (Table 2).

**Table 2:** **Bacterial genera showing significant differences in relative abundance (%) according to presence or absence of biochar**.

	**GENUS**	t1	t2	t3	t6	t9	t10	t12	t13
*A*	*Achromob acter*	−	−	−	−				
	*Dyella*	−	−	−					
	*Granulicella*	−		−					
	*Bordetella*		−	−					
	*Asticcacaulis*		+		−				
	*Aquincola*		+		+				
	*Byssovorax*		+		+				
	*Gemmata*		+			+			
	*Acidobacterium*	−	−						
	*Acidicapsa*	−		−	−	−			
	*Telmatobacter*	−		−	−				
	*Burkholderia*	−		−	−				
	*Sporocytophaga*			−	−				
	*Chthonomonas*			+		−			
*B*	*Telmatospirillum*			−		−	−		
	*Acidocella*	−			−	−	−	−	−
	*Aeromicrobium*	+			+	+		+	+
	*Mesorhizobium*	+			+	+		+	+
	*Nitratireductor*	+			+	+		+	+
	*Bradyrhizobium*				+	+	+	+	+
	*Rhodoplanes*				+	+	+	+	+
	*Alkanibacter*				+	+	+	+	+
	*Streptomyces*				+	+		+	+
	*Gemmatimonas*				+	+		+	+
	*Planctomyces*				+	+		+	+
	*Marmoricola*	+	+		+	+		+	
	*Rickettsia*				+	+	+	+	
	*Parvibaculum*				+	+		+	
	*Devosia*	+			+	+			
	*Verrucomicrobium*	+			−	−			
	*Holophaga*				+	+			
	*Woodsholea*				+	+			
	*Arthrobacter*				+			+	
	*Steroidobacter*				+			+	
	*Massilia*				−				+
	*Pseudomonas*				+				+
	*Pseudoxanthomonas*				+		−		
*C*	*Nocardioides*	+				+		+	+
	*Jatrophihabitans*					+	+	+	+
	*Crossiella*					+	+	+	+
	*Pseudolabrys*					+	+	+	+
	*Novosphingobium*					−		−	
	*Nitrosospira*					+		+	
	*Spirochaeta*					+		+	
	*Bryocella*					+	+		
	*Ferruginibacter*							+	+
	*Taibaiella*						+	+	+
	*Dongia*							+	+
	*Sediminibacterium*						+	+	
	Total +	7	5	1	21	23	10	24	18
	Total –	8	4	9	9	6	3	2	1

*Bacterial genera showing significant differences according to the presence of biochar are listed. Only those genera significantly different in relative abundance between the biochar and non-biochar treated peat for at least two time points are listed. Genera that decrease in relative abundance in the rhizosphere of biochar-treated peat compared to the control (non-biochar) are indicated with a “−” those that increase in relative abundance are indicated with a “+.” The total number of bacterial genera that increased or decreased in relative abundance are indicated at the bottom of the table*.

#### Bacterial and fungal richness

**Figure 2:**
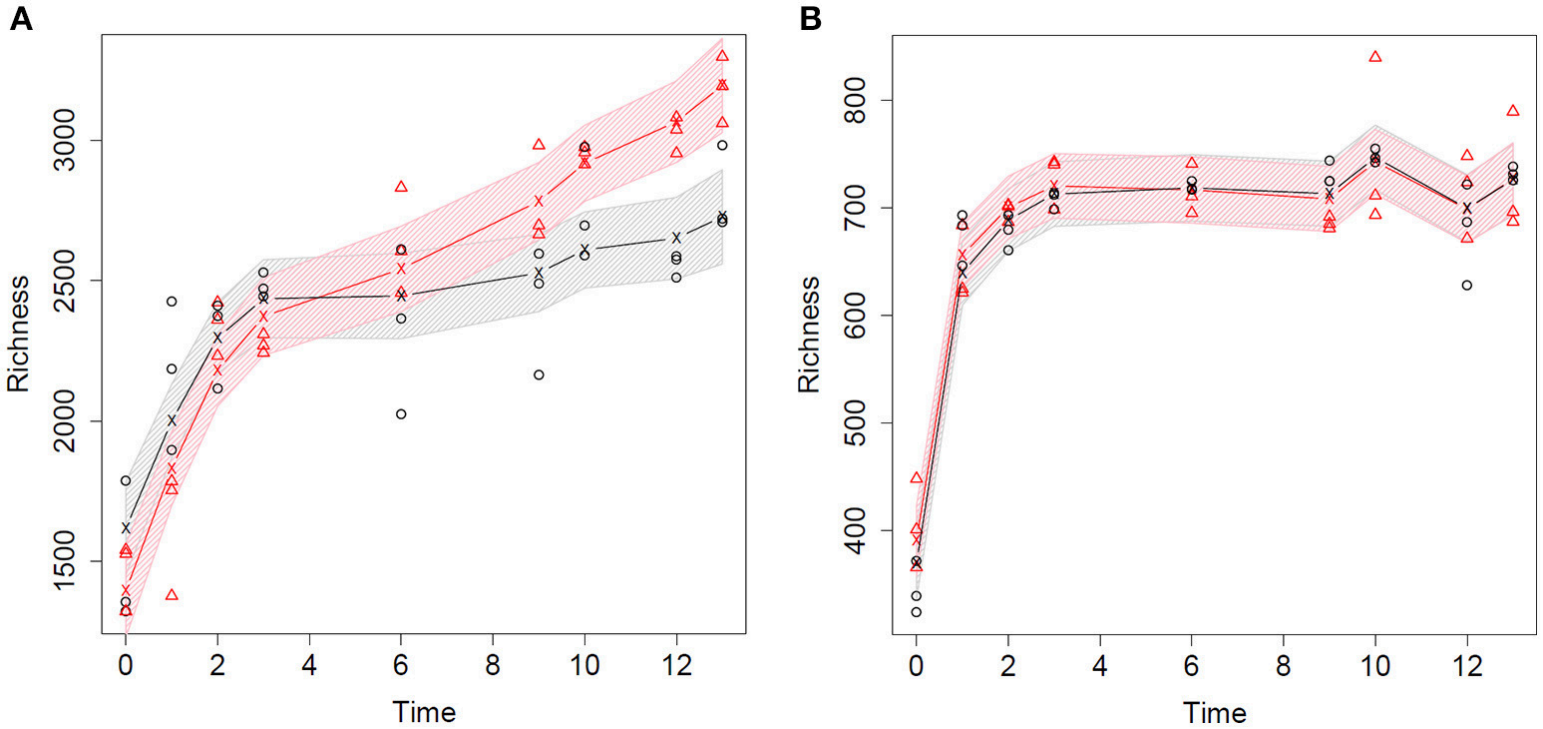
**Richness of the microbial community in the strawberry rhizosphere measured over 13 weeks of plant growth**. The richness derived from peat and biochar-amended samples are indicated in black and red, respectively. The observed rarified richness's of the biological replicates are depicted with dots. The lines represent the fitted average richness using an additive model with thin plate regression smoothers. The shaded areas are simultaneous 95% confidence bands that are estimated on a grid spanned by the observed time-points (*t* = 0, 1, 2, 3, 6, 9, 10, 12, 13 weeks). **(A)** Bacterial community richness of the strawberry rhizosphere. **(B)** Fungal community richness of the strawberry rhizosphere.

The bacterial and fungal community richness of the strawberry rhizosphere was estimated over 13 weeks of plant growth (Figure 2). After planting the cold-stored bare-root strawberry plants, a significant increase in bacterial richness is seen at time points 0, 1, and 2 (*p* < 0.05, Supplementary Figure 4A), for plants grown in peat and in peat amended with biochar. Subsequently, the bacterial richness continued increasing till the end of the strawberry growing period (week 13), although not significantly (Figure 2A; Supplementary Figure 4A). The number of fungal OTUs in the strawberry rhizosphere increased significantly at week 0 and 1 of plant growth (*p* << 0.01), but stabilized thereafter, independent whether biochar had been added to the peat (Figure 2B; Supplementary Figure 4B).

The average bacterial richness of the strawberry rhizosphere was significantly larger in the biochar amended peat than in the non-amended peat from week 9 of plant growth onwards (*p* < 0.01, Supplementary Figure 5A). Before week 9 the effect of biochar addition was not significant. In contrast, no significant effects of the addition of biochar were observed for the fungal rhizosphere richness (*p* > 0.65, Supplementary Figure 5B).

### *B. cinerea* leaf inoculation experiment

In this experiment, the effect of an above-ground infection (plant leaf) on the bacterial rhizosphere was studied. In addition, the effect of biochar addition to the peat and the interaction with the above-ground infection was studied. Rhizosphere samples were taken only at the end of plant growth (13 weeks of growth), 1 week after the inoculation with *B. cinerea*. Only the effects on the bacterial community were observed, as the time course experiment showed that the number and composition of fungal OTUs did not change from week three onwards and were not affected by the addition of biochar.

#### The bacterial rhizosphere community composition

**Figure 3:**
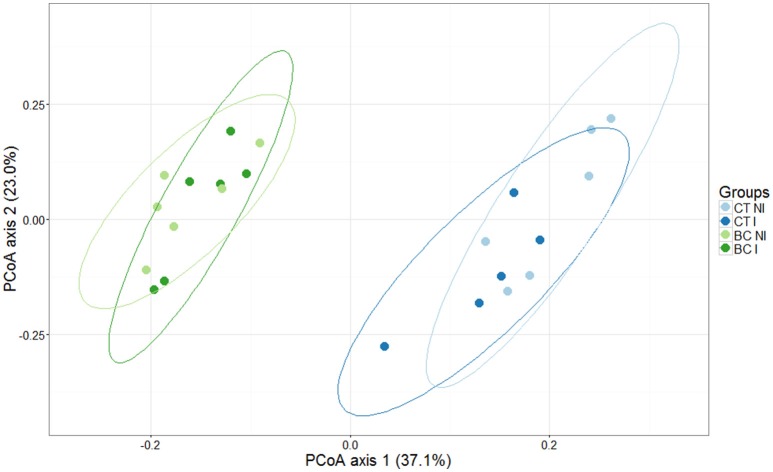
**Principal Coordinate Analysis (PCoA) profile of pairwise community dissimilarity (Bray-Curtis) indices of 16S V3–V4 sequencing data of the strawberry rhizosphere grown in biochar-amended (green) and unamended (blue) peat**. Ellipses represent the 95% confidence intervals. Half of the plants were infected (I) with *B. cinerea* (dark colored), the other half were not (NI) (light colored). The first and second axes represent 37.1% and 23.0% of the variance in the dataset, respectively. A clear separation is seen in the first axis, representing the major amount of variance in the dataset due to the biochar (BC) addition. Microbiome sequences of plants grown in non-biochar treated peat are indicated as control (CT).

The main effects of biochar addition and inoculation of the strawberry leaves with *B. cinerea* on the bacterial community composition of the strawberry rhizosphere and the interaction between both were studied using PERMANOVA analysis. This could be done because the condition of homogeneity of variances was fulfilled. The addition of biochar had a significant effect on the bacterial community (*p* < 0.001), but there was no general effect of infection and no interaction effect was revealed. This is illustrated with a PCoA plot, in which the first axis seems to corresponds to the variability in the community composition due to the addition of biochar and the second axis to the percentage of variability due to infection (Figure 3).

Subsequently, four individual comparisons were made, studying: (1) the effect of biochar on the rhizosphere bacterial community of non-inoculated plants, (2) the effect of biochar on the rhizosphere bacterial community of inoculated plants, (3) the effect of *B. cinerea* inoculation on the rhizosphere bacterial community of strawberry plants grown in peat, and (4) the effect of *B. cinerea* inoculation on the rhizosphere bacterial community of strawberry plants grown in biochar-amended peat. For these analyses, the OTU table was clustered on genus level. For non-infected plants, the 13 weeks of growth in substrate with biochar had a significant influence on the relative bacterial abundances. In total, 38 bacterial genera increased and 25 bacterial genera decreased in relative abundance when compared to the rhizosphere microbiome of the plants grown in the unamended peat (Table S3). The effect of biochar was less prominent when plants were infected: 12 bacterial genera increased and 17 decreased in relative abundance when compared to the rhizosphere microbiomes of the infected plants grown in the unamended peat (Table S3).

Second, the effect of infection on the bacterial community composition was studied. In total, 31 bacterial genera increased and three genera decreased significantly in relative abundance in the strawberry-peat bio-assay due to inoculation of the leaves with *B. cinerea*. The above-ground infection did not alter the bacterial community of the rhizosphere of strawberry grown in biochar amended peat (Table 3).

**Table 3:** **Significant differences in the relative abundance of bacterial genera (%) ± standard error between strawberry rhizospheres in peat with and without infection of ***B. cinerea*** on the strawberry leaves (***n*** = 6)**.

**Phylum**	**Family**	**Genus**	**Peat—NI**	**Peat—I**	**Peat + BC—NI**	**Peat + BC—I**
Acidobacteria	Unknown Family	*Bryobacter*	2.13±0.23	1.09±0.09^*^	0.64 ± 0.04	0.71 ± 0.04
	Acidobacteriaceae	*Edaphobacter*	0.50±0.04	0.29±0.04^*^	0.28 ± 0.02	0.31 ± 0.02
Actinobacteria	Acidothermaceae	*Acidothermus*	0.33±0.04	0.84±0.14^*^	0.77 ± 0.09	0.92 ± 0.09
	Cellulomonadaceae	*Cellulomonas*	<0.01	0.06±0.06^*^	< 0.01	0.01 ± 0.00
	Conexibacteraceae	*Conexibacter*	0.13±0.02	0.38±0.05^*^	0.23 ± 0.02	0.30 ± 0.03
	Frankiaceae	*Jatrophihabitans*	0.06±0.01	0.14±0.06^*^	0.34 ± 0.03	0.39 ± 0.04
	Iamiaceae	*Iamia*	< 0.01	0.02±0.01^*^	0.01 ± 0.01	0.01 ± 0.00
	Intrasporangiaceae	*Phycicoccus*	< 0.01	0.04±0.01^*^	0.02 ± 0.01	0.08 ± 0.05
	Micromonosporaceae	*Actinoplanes*	0.00±0.00	0.01±0.01^*^	< 0.01	0.03 ± 0.03
	Mycobacteriaceae	*Mycobacterium*	0.06±0.01	0.26±0.02^*^	0.30 ± 0.03	0.30 ± 0.05
	Nocardiaceae	*Nocardia*	0.03±0.01	0.08±0.02^*^	0.06 ± 0.01	0.08 ± 0.01
		*Williamsia*	< 0.01	0.08±0.07^*^	< 0.01	< 0.01
	Nocardioidaceae	*Aeromicrobium*	< 0.01± <0.0	0.02±0.01^*^	0.01 ± 0.00	0.03 ± 0.01
		*Marmoricola*	< 0.01	0.03±0.01^*^	0.02 ± 0.01	0.04 ± 0.01
		*Nocardioides*	0.05±0.01	0.19±0.05^*^	0.36 ± 0.03	0.45 ± 0.06
	Patulibacteraceae	*Patulibacter*	< 0.01	0.02±0.01^*^	< 0.01	0.01 ± 0.00
	Pseudonocardiaceae	*Pseudonocardia*	0.01±0.00	0.03±0.01^*^	0.03 ± 0.00	0.03 ± 0.00
	Solirubrobacteraceae	*Solirubrobacter*	< 0.01	0.02±0.01^*^	0.02 ± 0.00	0.02 ± 0.00
	Streptomycetaceae	*Streptomyces*	0.01±0.00	0.05±0.05^*^	0.08 ± 0.02	0.11 ± 0.04
Armatimonadetes	Chthonomonadaceae	*Chthonomonas*	1.31±0.34	0.38±0.17^*^	0.45 ± 0.06	0.46 ± 0.07
Bacteroidetes		*Flavobacterium*	0.02±0.01	0.16±0.10^*^	0.01 ± 0.00	0.01 ± 0.00
	Sphingobacteriaceae	*Pedobacter*	0.01±0.01	0.10±0.05^*^	< 0.01	0.01 ± 0.00
Planctomycetes	Planctomycetaceae	*Gemmata*	0.25±0.02	0.47±0.10^*^	0.50 ± 0.04	0.50 ± 0.02
		*Pirellula*	0.00±0.00	0.01±0.01^*^	0.01 ± 0.00	0.01 ± 0.01
		*Planctomyces*	0.02±0.00	0.04±0.00^*^	0.11 ± 0.01	0.10 ± 0.01
		*Singulisphaera*	0.03±0.00	0.06±0.01^*^	0.07 ± 0.00	0.07 ± 0.00
Proteobacteria	Bradyrhizobiaceae	*Rhodopseudomonas*	0.01±0.00	0.02±0.02^*^	0.05 ± 0.01	0.05 ± 0.00
	Hyphomicrobiaceae	*Devosia*	0.44±0.07	0.78±0.18^*^	0.96 ± 0.05	1.12 ± 0.08
	Hyphomonadaceae	*Hirschia*	0.01±0.01	0.09±0.04^*^	0.02 ± 0.01	0.02 ± 0.00
		*Woodsholea*	< 0.01±0.00	0.02±0.02^*^	0.04 ± 0.01	0.03 ± 0.01
	Phyllobacteriaceae	*Mesorhizobium*	0.01±0.01	0.04±0.01^*^	0.04 ± 0.01	0.05 ± 0.02
		*Nitratireductor*	0.01±0.001	0.04±0.01^*^	0.05 ± 0.01	0.06 ± 0.00
	Rhizobiaceae	*Shinella*	0.02±0.01	0.07±0.05^*^	0.06 ± 0.01	0.07 ± 0.02
	Rhodospirillaceae	*Defluviicoccus*	< 0.01	0.01±0.01^*^	< 0.01	< 0.01

*Genera followed by an asterisk indicate a significant increase or decrease in the relative abundance in the infected samples as compared to the non-infection samples for the non-biochar treatment. As a comparison, the values of the biochar treated samples are included in the table in gray, in which no significant differences were observed. NI = non-infected, I = infected, BC = biochar addition*.

#### Bacterial richness

The number of OTUs was estimated for each of the four groups (peat NI, peat I, peat+BC NI, and peat+BC I). For strawberry plants grown in peat, the average bacterial richness of the rhizosphere was 1327.5 ± 9.0 and 1509.3 ± 73.8 OTUs for the non-infected and the *B. cinerea* infected plants, respectively. For strawberry plants grown in biochar-amended peat, the average bacterial richness was 1698.1 ± 32.1 and 1654.2 ± 38.9 OTUs for the non-infected and the *B. cinerea* infected plants respectively (Supplementary Figure 6). In general, two significant effects were measured. First, infection of the plants induced a significant increase in bacterial richness (*p* = 0.039) in the rhizosphere when the plants were grown in unamended peat. Second, biochar amended to the peat substrate also induced a significant increase in bacterial richness (*p* < 0.01) in the rhizosphere, and this condition was not further influenced by the infection of the plants.

## Discussion

In previous studies, the effect of biochar on the microbial community of the rhizosphere was analyzed on a single sampling date in pot trials (Kolton et al., 2011; De Tender et al., 2016; Egamberdieva et al., 2016). In the present study, we have evaluated the temporal dynamics of bacterial and fungal communities of the strawberry rhizosphere by sampling at nine time points during the 13 week strawberry growing period. Two factors were evaluated: (1) the change in the rhizosphere microbiome community over time, and (2) the effect of biochar addition to peat on the bacterial and fungal community of the strawberry rhizosphere. First, we showed that the bacterial and fungal community changed in composition and richness over time. The fungal community composition changed mainly during the first week of plant growth and stabilized thereafter, with the exception of the Auriculariales. In contrast, the composition of the bacterial community changed especially during the first 6 weeks of plant growth. We believe that the major shift in bacterial and fungal community composition in the rhizosphere, that occurs in the first week of plant growth, is mediated by a change in growing medium. At the beginning of the experiment, the growing medium of the strawberry plants changed from field soil (nursery fields) to peat, which is common practice in Belgian strawberry cultivation (Lieten et al., 1995; Lieten, 2013). One of the main drivers of the rhizosphere microbiome is soil type (Berg and Smalla, 2009). Therefore, changes brought in this bulk soil will eventually affect the rhizosphere microbial community (Mendes et al., 2014). During the experiment, no significant changes in chemical parameters of the peat were observed over time. This indicates that the shift in bacterial community profile of the rhizosphere measured from week 1 to 6 was not mediated by a shift in chemical composition of the growing medium. Within the experiment, plants were either in a vegetative phase (week 1–3) or in a flowering and fruiting stage (from week 4 onwards). It has already been shown that the rhizosphere composition could be different between plant stages and that this could be related to the excretion of chemical compounds through the roots (Chaparro et al., 2014). We therefore hypothesize that the observed changes in the first weeks of plant growth were plant-driven, as plants can influence the rhizosphere by the release of rhizodeposits from the roots (Philippot et al., 2013). An alternative hypothesis could be that the rhizosphere microbiome is influenced by an interplay between the microorganisms of the plant root itself (Dennis et al., 2010).

Second, the effect of biochar on the bacterial and fungal community of the rhizosphere was evaluated over time. During the strawberry growth period, no effect on the fungal rhizosphere community was observed due to the addition of biochar. In contrast, starting at week 9 of plant growth onwards, increased richness was observed in the biochar-treated bacterial rhizosphere community as compared to the non-biochar treated rhizosphere. Additionally, a higher number of genera were induced from week 6 onwards. Some of these genera contain species previously reported to be involved in the N-cycle, e.g., *Nitratireductor, Devosia, Nitrospira*, and *Taibaiella* (Rivas et al., 2002; Hoque et al., 2011; Penton et al., 2013; Zhang et al., 2013; Kox and Jetten, 2015) and some contain species that can act as biocontrol agent, e.g., *Streptomyces* and *Nocardioides* (Carrer et al., 2008; Saharan and Nehra, 2011; Viaene et al., 2016). These so-called plant-growth promoting rhizobacteria (PGPR) have been extensively studied because of their beneficial effects on plant growth and health (Berendsen et al., 2012). This is in accordance with previous research in which we studied the rhizosphere community at the end of the strawberry growth period in peat and also found PGPR linked genera which increased by biochar addition to peat (De Tender et al., 2016). Based on the results of the current study, we believe that the effect of biochar on the rhizosphere bacterial community is postponed, as it is measurable from week 6 of strawberry growth.

To our knowledge, this is the first time the temporal variability on the strawberry rhizosphere bacterial and fungal community has been measured. Additionally, it is the first time that the effect of biochar on the rhizosphere community has been measured in time during the entire growth cycle of the plant.

Within the time course experiment and the inoculation experiment, the effect of biochar on some plant properties after 13 weeks of plant growth was evaluated. The addition of biochar to fertilized and limed peat resulted in an increase in plant root development and strawberry fruit production. We suggest the following explanations: (1) biochar could have a nutrient-addition effect, i.e., could act as a fertilizer in the growing medium. In previous experiments biochar addition to unfertilized peat resulted in an increase in plant growth (De Tender et al., 2016). These effects however were less obvious once the peat was fertilized, indicating a role of fertilization on the effect of biochar on strawberry growth (De Tender et al., 2016). Those experiments also revealed that incorporating biochar to peat resulted in an increase in potassium (K) concentration of 20.65 mg L^−1^ peat to 36.15 mg L^−1^ in unfertilized peat and 93.40–108.0 mg L^−1^ in fertilized peat. Strawberry has high nutrient demands, especially for K (Tagliavini et al., 2005). A potential higher K concentration in the growing medium may have resulted in the higher production of strawberry fruits, as K has an effect on fruit quality and quantity, and roots in the biochar treatment (Ebrahimi et al., 2012). To prove this concept however, more research is necessary in which the concentration of K and other nutrients should be measured in the strawberry plant and fruits. (2) The effect of biochar on the plant properties could be indirect through a change in the rhizosphere microbiome. The increase in PGPR bacteria from week 6 onwards, could have resulted in the higher production of strawberry fruits and roots in the biochar treatment. (3) Biochar could have a direct or indirect (through its effect on the rhizosphere microbiome) response on the auxin pathway of the plant. It has been shown that biochar application can induce auxin-related genes (Viger et al., 2014). Auxin is known to be connected with the plant root development, with a major role in the production of lateral roots and root hairs (Overvoorde et al., 2010). Subsequently, it is known that bacteria interacting with the plant, i.e., rhizosphere associated organisms, can produce auxin and interfere with the auxin-regulated plant developmental processes (Spaepen and Vanderleyden, 2011). Therefore, the increase in lateral root development of the plants grown in biochar-amended peat could be auxin-related.

Biochar also increased the post-harvest resistance of the strawberry fruits against *B. cinerea*. Previously, it has been shown that biochar increased disease resistance of (1) strawberry grown in peat against *B. cinerea, Colletotrichum acutatum*, and *Podosphaera apahanis* leaf infections (Meller Harel et al., 2012) and (2) field-grown pepper and tomato plants against to *B. cinerea* and *Leveillula taurica* leaf infections (Elad et al., 2010). Moreover, induced resistance against soilborne pathogens, including nematodes, by biochar has been reported more recently (Huang et al., 2015; Jaiswal et al., 2015; George et al., 2016). Following hypotheses are made for the mechanisms involved: (1) A nutrient addition effect of biochar (see above), as the susceptibility of plants to diseases is also known to be influenced by its nutritional status (Nam et al., 2006; Lecompte et al., 2010; Xu et al., 2013). Therefore, in further research the plant tissue nutrient content should be analyzed in the biochar-amended plants as compared to the control plants, (2) The change in the rhizosphere microbiome from week six onwards due to biochar toward bacteria involved in biological control. It is known that the susceptibility of plants to diseases is influenced by its rhizosphere microbiome (e.g., Berendsen et al., 2012), (3) Biochar could have directly or indirectly (through its effect of the rhizosphere microbiome) changed the metabolite composition of the strawberry fruits. It is shown that biochar addition to substrate alters the secondary metabolite composition of tomato fruits (Petruccelli et al., 2015). Furthermore, antioxidant and fatty acid composition of fruits has been shown to be related to the disease resistance of fruits (Cao et al., 2014). Therefore, biochar addition to the peat, could result in a change in strawberry fruit composition, which could make them less susceptible for *B. cinerea* infection. This effect of biochar on the overall composition of the strawberry fruits will be tested in future experiments, based on the protocol described in Palencia et al. (2016).

We established an experiment to evaluate the effect of *B. cinerea* on the strawberry rhizosphere microbiome after leaf inoculation on plants grown in peat with and without incorporation of biochar. First we observed an increase in bacterial richness when strawberry plants grown in peat were infected with *B. cinerea*; this was not observed in plants grown in peat amended with biochar. The microbial species richness of the rhizosphere has been proposed as a predictor of the above-ground plant diversity and productivity (van der Heijden et al., 2008; Lau and Lennon, 2011; Wagg et al., 2011). A higher belowground richness and diversity could even act as an insurance for maintaining plant productivity, even under changing environmental conditions (Wagg et al., 2011). Therefore, the increase in bacterial richness could be a reaction of the plant on the above-ground infection, to maintain its productivity or counteract the pathogen. Second, the relative abundance of 34 bacterial genera in the strawberry rhizosphere was influenced by the above-ground infection in the non-biochar treatment, but was not seen in the biochar-amended peat. It is known that five factors can influence the rhizosphere microbial composition: soil type, plant genotype, addition of fertilizers, crop rotation and application of pesticides (Massart et al., 2015). Rosberg et al. (2014) already showed that inoculation of tomato with the root pathogen *Pythium* results in changes in the rhizosphere community. Nonetheless, to our knowledge the present study is the first report to reveal that an above-ground fungal infection can change the rhizosphere community composition. Additionally, this effect seemed to be neutralized once biochar is added to the peat. A comparison of the relative abundances of the bacterial genera that were significantly changed in the peat-assay compared to biochar-amended peat, reveals that after infection the relative abundance of the bacterial genera came near the levels observed in the rhizosphere of the biochar-amended peat for the same genera. This indicates that biochar already “prepared” the rhizosphere's community to the infection in three ways: (1) by increasing the richness of the bacterial community, (2) by shifting toward a higher relative abundance of genera including species acting as biocontrol agent or involved in the N-cycle, and (3) by shifting the relative abundance of the bacterial genera in the rhizosphere toward those which we obtain after infection of the plant. However, these rhizosphere microbiome effects were not accompanied with an increased resistance of the leaves against *B. cinerea*. This may be attributed to the low disease severity observed on the leaves (about 15% of the leaf area was affected in the control treatment). Previous research of De Tender et al. (2016) showed that biochar amendment in unfertilized and non-limed peat increased the resistance of leaves against *B. cinerea*, but in that experiment a disease severity of 50% was observed in the control treatment. Using more conducive conditions for *B. cinerea* on the leaves in the present study may have shown a difference between both treatments, but further research is necessary to confirm this.

In conclusion, this paper indicates that upon both biochar incorporation in peat and above ground pathogen attack, plants recruit rhizosphere microbes that may help them in their defense and plant growth promotion. These findings are important for a sustainable strawberry production worldwide.

## Author contributions

CD, MM, and JD were involved in the design and the supervision of the experiments. CD, PC, and JD conducted the strawberry plant experiments. BV and PC conducted the chemical analysis of the peat substrates. CD, PD, and AH conducted the amplicon sequencing and the bio-informatics of the NGS data. CD and LC conducted the statistical analysis of the NGS data. CD wrote the first draft and finalized the manuscript. All authors contributed to the writing of the manuscript and approved submission.

### Conflict of interest statement

The authors declare that the research was conducted in the absence of any commercial or financial relationships that could be construed as a potential conflict of interest.
