# Supplementary material for: Dynamics in the Strawberry Rhizosphere Microbiome in Response to Biochar and Botrytis cinerea Leaf Infection
Source: Front Microbiol. 2016 Dec 22;7:2062. doi: 10.3389/fmicb.2016.02062 (PMC5177642; doi:10.3389/fmicb.2016.02062)
Supplement: Supplementary file 1 [file DataSheet1.DOCX]

**Table S1**: Chemical properties of peat and peat amended with 3% biochar during a 13 weeks interval. Values are averages±standard errors for 3 replicates

|  | **pH-H_2_O** | | **EC**  **(µS/cm)** | | **NO_3_-N**  **(mg/L peat)** | | **NH_4_-N**  **(mg/L peat)** | | **SO_4_**  **(mg/L peat)** | | **Cl**  **(mg/L peat)** | | **P in H_2_O**  **(mg/L peat)** | |
| --- | --- | --- | --- | --- | --- | --- | --- | --- | --- | --- | --- | --- | --- | --- |
|  | **Peat (a)** | **peat+BC (b)** | **peat** | **Peat+BC** | **peat** | **Peat+BC** | **Peat (a)** | **Peat+BC (b)** | **peat** | **Peat+BC** | **peat** | **Peat+BC** | **peat** | **Peat+BC** |
| **Optimal range** | **3.8 – 6.0** | | **200 - 400** | | **Not applicable** | | **Not applicable** | | **0-100** | | **0-100** | | **Not applicable** | |
| T1 | 4.41±0.06 | 4.73±0.07 | 76.33±8.83 | 68.67±5.33 | 6.50±1.21 | 6.60±0.90 | 10.83±1.85 | 6.73±1.14 | 36.17±9.73 | 38.40±5.12 | 10.00±0.00 | 10.23±0.12 | 5.93±1.04 | 5.43±0.68 |
| T2 | 4.83±0.32 | 4.79±0.04 | 91.00±27.05 | 70.00±8.62 | 9.70±3.60 | 6.90±0.71 | 16.43±7.38 | 5.03±0.03 | 56.63±30.41 | 30.60±0.61 | 10.37±0.37 | 10.77±0.39 | 8.63±3.93 | 4.80±0.10 |
| T3 | 4.73±0.09 | 5.04±0.20 | 37.00±10.00 | 55.00±9.64 | 5.00±0.00 | 7.67±2.67 | 5.87±0.87 | 5.07±0.07 | 12.50±0.80 | 24.00±3.98 | 10.70±0.70 | 10.00±0.00 | 4.70±0.00 | 5.10±0.40 |
| T6 | 4.35±0.05 | 4.90±0.03 | 86.33±16.68 | 46.67±3.17 | 8.97±2.18 | 5.93±0.66 | 6.97±1.97 | 5.00±0.00 | 49.93±15.86 | 23.17±4.4 | 11.40±0.84 | 10.00±0.00 | 8.50±2.06 | 4.70±0.00 |
| T9 | 4.28±0.10 | 4.61±0.06 | 83.00±43.25 | 50.33±6.96 | 13.57±8.57 | 5.00±0.00 | 9.73±4.73 | 5.00±0.00 | 50.73±32.09 | 35.03±9.09 | 13.23±3.23 | 10.57±0.43 | 10.10±5.40 | 4.70±0.00 |
| T10 | 4.41±0.05 | 5.18±0.58 | 80.67±7.86 | 59.00±14.01 | 6.17±1.17 | 7.13±2.13 | 6.37±1.37 | 5.00±0.00 | 45.07±12.88 | 34.37±8.94 | 18.43±6.74 | 13.03±2.84 | 6.00±1.30 | 5.47±0.77 |
| T12 | 4.36±0.02 | 4.72±0.04 | 61.67±8.84 | 49.00±4.51 | 5.47±0.37 | 5.00±0.00 | 5.60±0.60 | 5.00±0.00 | 31.50±7.04 | 32.70±5.24 | 14.83±3.36 | 10.10±0.10 | 4.83±0.13 | 4.70±0.00 |
| T13 | 4.36±0.02 | 4.75±0.09 | 51.68±2.91 | 51.67±7.36 | 5.00±0.00 | 5.23±0.27 | 5.60±0.00 | 5.00±0.00 | 26.63±3.70 | 39.47±7.91 | 10.00±0.00 | 11.03±0.61 | 4.70±0.00 | 4.70±0.00 |

**Table S2**: Chemical properties of peat and peat amended with 3% biochar after 13 weeks of plant growth. Part of the plants were infected with *B. cinerea*. Values are averages±standard errors for 10 replicates. (NI= not infected, I= leafs infected with *B. cinerea*)

|  | **pH-H_2_O** | **EC**  **(µS/cm)** | **NO_3_-N**  **(mg/L peat)** | **NH_4_-N**  **(mg/L peat)** | **SO_4_**  **(mg/L peat)** | **Cl**  **(mg/L peat)** | **P in H_2_O**  **(mg/L peat)** |
| --- | --- | --- | --- | --- | --- | --- | --- |
| **Optimal range** | **3.8-6.0** | **200-400** | **Not applicable** | **Not applicable** | **0-100** | **0-100** | **Not applicable** |
| Peat - NI | 4.31±0.04 (a) | 76.00±17.90 (a) | 8.24±2.49 (a) | 6.61±1.61 (a) | 47.24±14.45 (a) | 10.66±0.66 (a) | 8.15±3.38 (a) |
| Peat - I | 4.55±0.20 (ab) | 76.27±12.55 (a) | 6.45±0.87 (a) | 5.47±0.35 (a) | 42.98±9.69 (a) | 14.93±4.90 (a) | 5.82±0.78 (a) |
| Peat + BC - NI | 4.86±0.05 (b) | 71.73±6.88 (a) | 8.14±1.67 (a) | 5.12±0.08 (a) | 58.09±6.15 (a) | 12.15±0.72 (a) | 6.30±0.69 (a) |
| Peat + BC - I | 4.84±0.05 (b) | 59.58±7.28 (a) | 7.51±1.67 (a) | 5.00±0.00 (a) | 47.07±6.98 (a) | 10.93±0.43 (a) | 5.48±0.81 (a) |

**Table S3:** Significant differences in the relative abundance of bacterial genera (%) ± standard error between strawberry rhizospheres in peat with and without 3% biochar (n = 6).

|  |  |  | **NOT INFECTED** | |  | **INFECTED** | |  |
| --- | --- | --- | --- | --- | --- | --- | --- | --- |
| **Phylum** | **Family** | **Genus** | **Peat** | **Peat + 3% BC** |  | **Peat** | **Peat + 3% BC** |  |
| Acidobacteria | Acidobacteriaceae (subgroup 1) | *Acidobacterium* | 1.15±0.07 | 0.59±0.04 | * | 1.04±0.08 | 0.61±0.05 | * |
| Acidobacteria | Acidobacteriaceae (subgroup 1) | *Edaphobacter* | 0.50±0.04 | 0.28±0.02 | * | 0.29±0.02 | 0.31±0.02 |  |
| Acidobacteria | Acidobacteriaceae (subgroup 1) | *Granulicella* | 3.02±0.13 | 2.11±0.13 | * | 2.51±0.22 | 2.43±0.21 |  |
| Acidobacteria | Acidobacteriaceae (subgroup 1) | *Telmatobacter* | 0.05±0.02 | 0.00±0.00 | * | 0.04±0.01 | 0.01±0.00 | * |
| Acidobacteria | Unknown family | *Bryobacter* | 2.13±0.23 | 0.64±0.03 | * | 1.09±0.09 | 0.71±0.04 | * |
| Actinobacteria | Acidothermaceae | *Acidothermus* | 0.33±0.04 | 0.78±0.09 | * | 0.84±0.06 | 0.92±0.09 |  |
| Actinobacteria | Conexibacteriaceae | *Conexibacter* | 0.13±0.02 | 0.23±0.02 | * | 0.38±0.05 | 0.30±0.03 |  |
| Actinobacteria | Frankiaceae | *Jatrophihabitans* | 0.06±0.01 | 0.34±0.03 | * | 0.14±0.03 | 0.39±0.04 | * |
| Actinobacteria | Iamiaceae | *Iamia* | 0.00±0.00 | 0.01±0.00 | * | 0.02±0.01 | 0.01±0.00 |  |
| Actinobacteria | Intrasporangiaceae | *Phycicoccus* | 0.00±0.00 | 0.02±0.00 | * | 0.03±0.01 | 0.08±0.04 |  |
| Actinobacteria | Microbacteriaceae | *Amnibacterium* | 0.00±0.00 | 0.01±0.00 | * | 0.01±0.000 | 0.01±0.00 |  |
| Actinobacteria | Mycobacteriaceae | *Mycobacterium* | 0.06±0.01 | 0.30±0.03 | * | 0.26±0.01 | 0.30±0.05 |  |
| Actinobacteria | Nakamurellaceae | *Nakamurella* | 0.00±0.00 | 0.01±0.00 | * | 0.01±0.00 | 0.02±0.01 |  |
| Actinobacteria | Nocardiaceae | *Nocardia* | 0.03±0.00 | 0.06±0.01 | * | 0.08±0.01 | 0.08±0.01 |  |
| Actinobacteria | Nocardioidaceae | *Aeromicrobium* | 0.00±0.00 | 0.01±0.00 | * | 0.01±0.00 | 0.03±0.01 |  |
| Actinobacteria | Nocardioidaceae | *Marmoricola* | 0.00±0.00 | 0.02±0.00 | * | 0.02±0.01 | 0.04±0.01 |  |
| Actinobacteria | Nocardioidaceae | *Nocardioides* | 0.05±0.01 | 0.36±0.03 | * | 0.19±0.05 | 0.45±0.06 | * |
| Actinobacteria | Pseudonocardiaceae | *Pseudonocardia* | 0.01±0.00 | 0.02±0.00 | * | 0.02±0.00 | 0.03±0.01 |  |
| Actinobacteria | Solirubrobacteraceae | *Solirurobacter* | 0.00±0.00 | 0.02±0.00 | * | 0.02±0.00 | 0.02±0.00 |  |
| Actinobacteria | Sterptomycetaceae | *Streptomyces* | 0.01±0.00 | 0.07±0.02 | * | 0.05±0.02 | 0.11±0.04 |  |
| Armatimonadetes | Chthonomonadales | *Chthonomonas* | 1.31±0.34 | 0.45±0.06 | * | 0.38±0.17 | 0.46±0.07 |  |
| Bacteroidetes | Chitinophagaceae | *Chitinophaga* | 0.15±0.03 | 0.08±0.03 |  | 0.16±0.02 | 0.04±0.02 | * |
| Bacteroidetes | Chitinophagaceae | *Sediminibacterium* | 0.08±0.02 | 0.47±0.14 | * | 0.21±0.12 | 0.28±0.07 |  |
| Bacteroidetes | Cytophagaceae | *Sporocytophaga* | 0.05±0.01 | 0.04±0.01 |  | 0.07±0.01 | 0.03±0.01 | * |
| Bacteroidetes | Flavobacteriaceae | *Flavobacterium* | 0.02±0.01 | 0.01±0.00 |  | 0.16±0.10 | 0.01±0.00 | * |
| Bacteroidetes | sphingobacteriaceae | *Mucilaginibacter* | 4.29±0.39 | 6.89±0.41 | * | 3.85±0.14 | 5.99±0.29 | * |
| Bacteroidetes | sphingobacteriaceae | *Pedobacter* | 0.01±0.01 | 0.00±0.00 |  | 0.10±0.05 | 0.01±0.00 | * |
| Gemmatimonadetes | Gemmatimonadaceae | *Gemmatimonas* | 0.01±0.01 | 0.05±0.01 | * | 0.04±0.02 | 0.05±0.01 |  |
| Planctomycetes | Planctomycetaceae | *Gemmata* | 0.25±0.02 | 0.50±0.04 | * | 0.47±0.05 | 0.50±0.02 |  |
| Planctomycetes | Planctomycetaceae | *Isosphaera* | 0.12±0.01 | 0.07±0.01 | * | 0.12±0.01 | 0.06±0.01 | * |
| Planctomycetes | Planctomycetaceae | *Pirellula* | 0.00±0.00 | 0.01±0.00 | * | 0.01±0.01 | 0.01±0.01 |  |
| Planctomycetes | Planctomycetaceae | *Planctomyces* | 0.02±0.00 | 0.11±0.01 | * | 0.04±0.01 | 0.10±0.01 | * |
| Planctomycetes | Planctomycetaceae | *Schlesneria* | 0.15±0.01 | 0.40±0.03 | * | 0.20±0.03 | 0.37±0.04 | * |
| Planctomycetes | Planctomycetaceae | *Singulisphaera* | 0.03±0.00 | 0.07±0.00 | * | 0.06±0.00 | 0.07±0.00 |  |
| Proteobacteria | Acetobacteraceae | *Acidicaldus* | 0.81±0.04 | 0.23±0.02 | * | 0.72±0.05 | 0.24±0.02 | * |
| Proteobacteria | Acetobacteraceae | *Acidiphilium* | 0.07±0.01 | 0.03±0.00 | * | 0.05±0.01 | 0.04±0.00 |  |
| Proteobacteria | Alcaligenaceae | *Achromobacter* | 0.10±0.02 | 0.07±0.03 |  | 0.12±0.03 | 0.03±0.01 | * |
| Proteobacteria | Beijerinckiaceae | *Methylocella* | 0.22±0.03 | 0.12±0.01 | * | 0.25±0.03 | 0.12±0.02 | * |
| Proteobacteria | Bradyrhizobiaceae | *Rhodopseudomonas* | 0.01±0.00 | 0.05±0.01 | * | 0.02±0.01 | 0.05±0.00 | * |
| Proteobacteria | Caulobacteraceae | *Caulobacter* | 0.11±0.01 | 0.05±0.00 | * | 0.14±0.03 | 0.05±0.00 | * |
| Proteobacteria | Coxiellaceae | *Aquicella* | 0.20±0.03 | 0.12±0.01 | * | 0.14±0.02 | 0.13±0.02 |  |
| Proteobacteria | Coxiellaceae | *Coxiella* | 0.07±0.01 | 0.01±0.00 | * | 0.04±0.02 | 0.02±0.01 | * |
| Proteobacteria | Cystobacteraceae | *Anaeromyxobacter* | 0.32±0.03 | 0.20±0.01 | * | 0.33±0.03 | 0.17±0.04 | * |
| Proteobacteria | Hyphomicrobiaceae | *Devosia* | 0.44±0.07 | 0.96±0.05 | * | 0.78±0.08 | 1.12±0.08 |  |
| Proteobacteria | Hyphomicrobiaceae | *Hyphomicrobium* | 0.01±0.00 | 0.04±0.01 | * | 0.02±0.01 | 0.05±0.02 | * |
| Proteobacteria | Hyphomicrobiaceae | *Rhodoplanes* | 0.01±0.00 | 0.04±0.01 | * | 0.02±0.01 | 0.04±0.01 |  |
| Proteobacteria | Hyphomonadaceae | *Hirschia* | 0.01±0.01 | 0.02±0.01 |  | 0.09±0.04 | 0.02±0.00 | * |
| Proteobacteria | Hyphomonadaceae | *Woodsholea* | 0.01±0.00 | 0.04±0.01 | * | 0.02±0.01 | 0.03±0.01 |  |
| Proteobacteria | Nevskiaceae | *Hydrocarboniphaga* | 0.01±0.00 | 0.36±0.13 | * | 0.00±0.00 | 0.17±0.14 | * |
| Proteobacteria | Nevskiaceae | *Nevskia* | 0.37±0.07 | 0.70±0.09 | * | 0.55±0.07 | 0.64±0.18 |  |
| Proteobacteria | Phyllobacteriaceae | *Mesorhizobium* | 0.01±0.00 | 0.04±0.01 | * | 0.03±0.01 | 0.05±0.02 | * |
| Proteobacteria | Phyllobacteriaceae | *Nitratireductor* | 0.01±0.00 | 0.05±0.01 | * | 0.04±0.01 | 0.06±0.00 |  |
| Proteobacteria | Polyangiaceae | *Sorangium* | 0.63±0.04 | 0.42±0.03 | * | 0.49±0.0 | 0.47±0.04 |  |
| Proteobacteria | Pseudomonadaceae | *Pseudomonas* | 0.06±0.05 | 0.01±0.00 | * | 0.02±0.00 | 0.03±0.01 |  |
| Proteobacteria | Rhizobiaceae | *Shinella* | 0.02±0.01 | 0.06±0.01 | * | 0.07±0.02 | 0.07±0.02 |  |
| Proteobacteria | Rhizobiales incertae sedis | *Bauldia* | 0.19±0.10 | 0.34±0.06 |  | 0.11±0.04 | 0.30±0.05 | * |
| Proteobacteria | Rhodospirillaceae | *Dongia* | 0.02±0.01 | 0.12±0.03 | * | 0.06±0.04 | 0.10±0.03 |  |
| Proteobacteria | Rhodospirillaceae | *Inquilinus* | 0.23±0.03 | 0.11±0.01 | * | 0.15±0.01 | 0.10±0.02 |  |
| Proteobacteria | Rhodospirillaceae | *Telmatospirillum* | 0.61±0.11 | 0.14±0.02 | * | 0.56±0.16 | 0.13±0.06 | * |
| Proteobacteria | Sphingomonadaceae | *Sphingobium* | 0.27±0.11 | 0.08±0.01 | * | 0.16±0.04 | 0.12±0.04 |  |
| Proteobacteria | Xanthobacteraceae | *Labrys* | 0.03±0.01 | 0.07±0.01 | * | 0.04±0.01 | 0.06±0.01 |  |
| Proteobacteria | Xanthobacteraceae | *Pseudolabrys* | 0.23±0.05 | 0.56±0.06 | * | 0.32±0.03 | 0.67±005 | * |
| Proteobacteria | Xanthomonadaceae | *Dyella* | 0.84±0.11 | 0.43±0.03 | * | 0.59±0.09 | 0.42±0.06 |  |
| Verrucomicrobia | Chthoniobacteraceae | *Chthoniobacter* | 2.06±0.16 | 1.40±0.11 | * | 1.81±0.19 | 1.25±0.10 | * |
| Verrucomicrobia | Opitutaceae | *Opitutus* | 2.32±0.31 | 4.18±0.21 | * | 2.48±0.33 | 3.93±0.33 | * |
| Verrucomicrobia | Verrucomicrobiaceae | *Verrucomicrobium* | 0.09±0.02 | 0.17±0.02 | * | 0.13±0.02 | 0.19±0.02 |  |


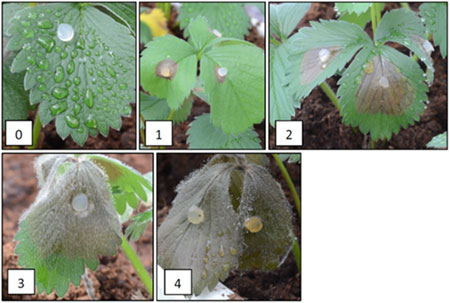


**Supplementary Figure 1**: Disease scale used for both the lettuce and strawberry leaf infection with Rhizoctonia solani and Botrytis cinerea respectively0 = 0% of the leaf area infected (no symptoms), 1 = < 25% of the leaf area is affected, 2 = 25% to 50% of the leaf area is affected, 3 = 51% to 75% of the leaf area is affected, 4 = >75% of the leaf area is affected.


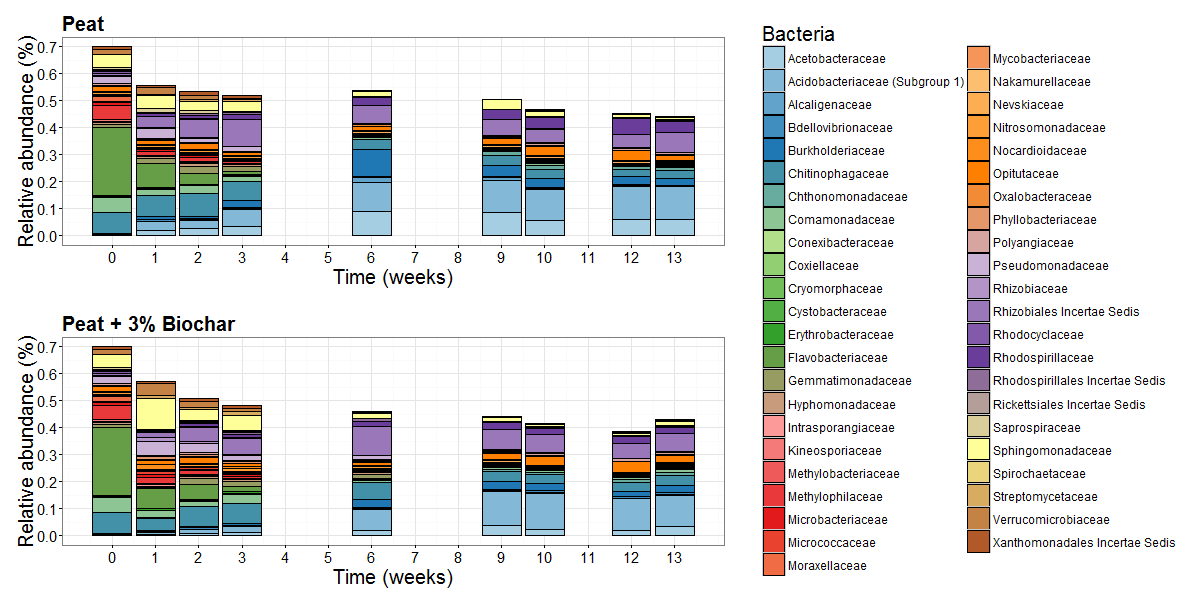


**Supplementary Figure 2: Representation of the bacterial families that change significantly in relative abundance (%) over time**. The mean relative abundance (n=3) of the bacterial families that change significantly over time between at least two successive time points are represented. Only those families that contribute for at least 0.1% of the total community for at least one time point are represented in the graph. Time points where no sampling was done are visualized as an empty space. Upper figure: The rhizosphere of strawberry grown in peat. Bottom figure: The rhizosphere of strawberry grown in peat amended with 3% biochar.


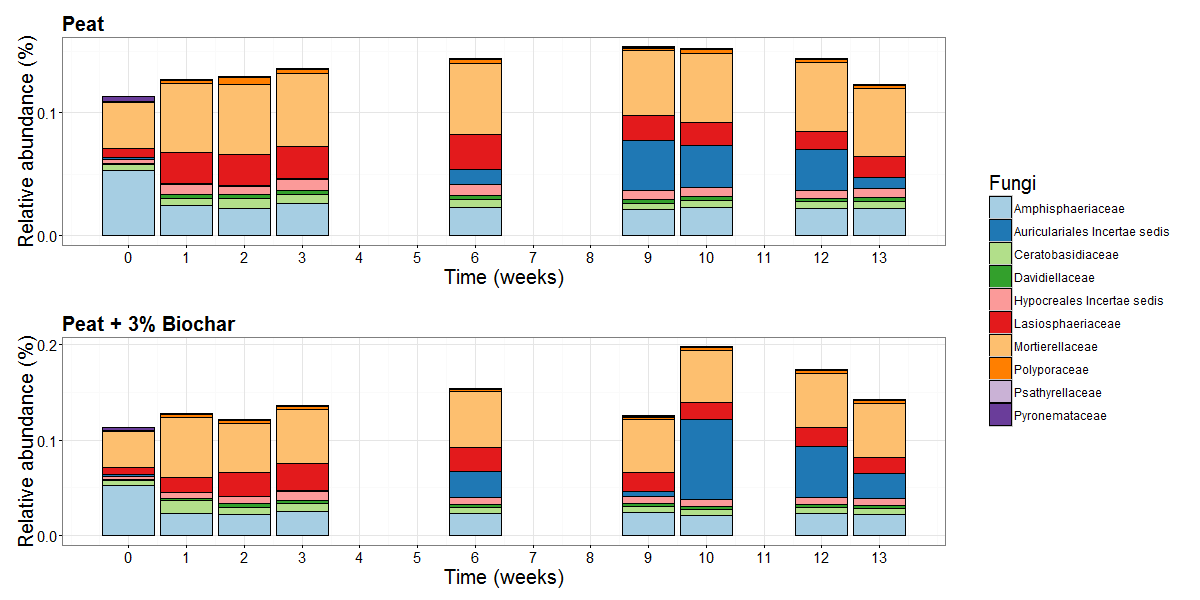


**Supplementary Figure 3:** **Representation of the fungal families that change significantly in relative abundance (%) over time**. The mean relative abundance (n=3) of the fungal families that change significantly over time between at least two successive time points are represented. Only those families that contribute for at least 0.1% of the total community for at least one time point are represented in the graph. Time points where no sampling was done are visualized as an empty space. Upper figure: The rhizosphere of strawberry grown in peat. Bottom figure: The rhizosphere of strawberry grown in peat amended with 3% biochar


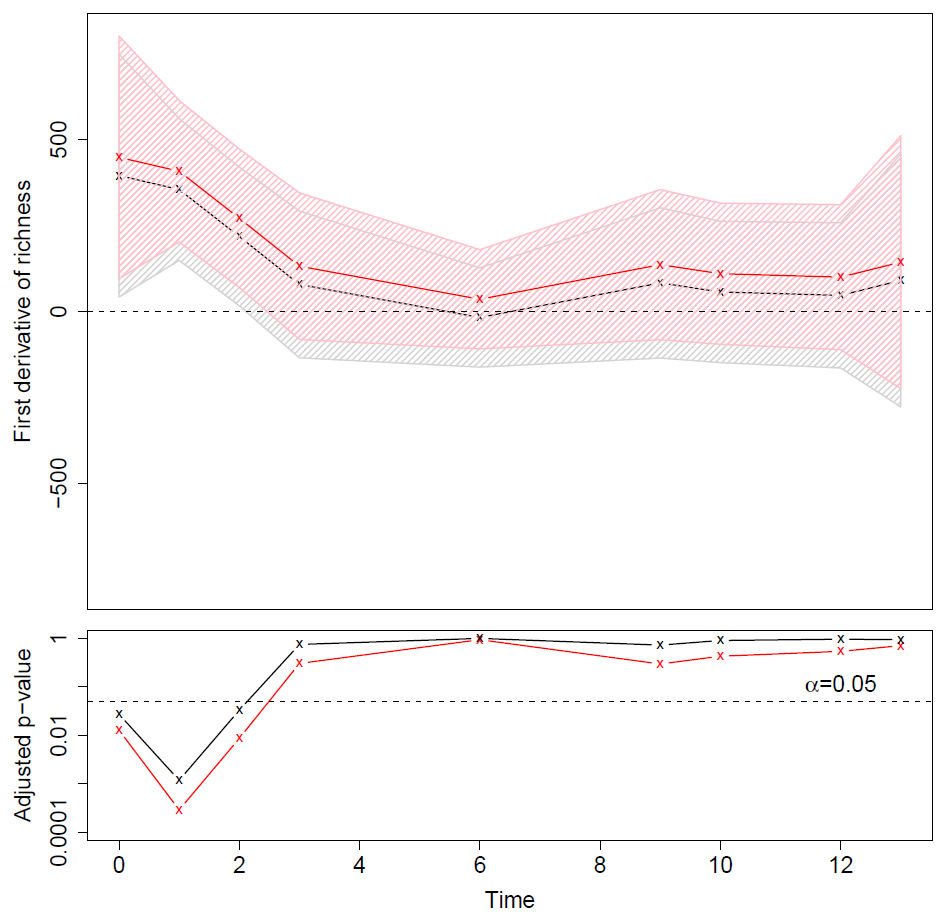


**Supplementary Figure 4A: First derivative of the richness of Bacteria in the strawberry rhizosphere over 13 weeks of plant growth.** The top panel depicts the first derivative for peat and biochar-amended richness profiles in black and red, respectively. The shaded areas are simultaneous 95 % confidence bands that are estimated on a grid spanned by the observed time-points (t=0, 1, 2, 3, 6, 9, 10, 12, 13 weeks). Both derivative profiles are higher for most time-points indicating that the richness is increasing over time. The highest increase is observed at time zero and the increase in richness for both peat and biochar amended media moderates as plant growth progresses. For both profiles the first derivative is significantly higher than zero at week 0, 1 and 2 at the 5% significance level. The bottom panel shows the corresponding p-value adjusted for multiple testing.


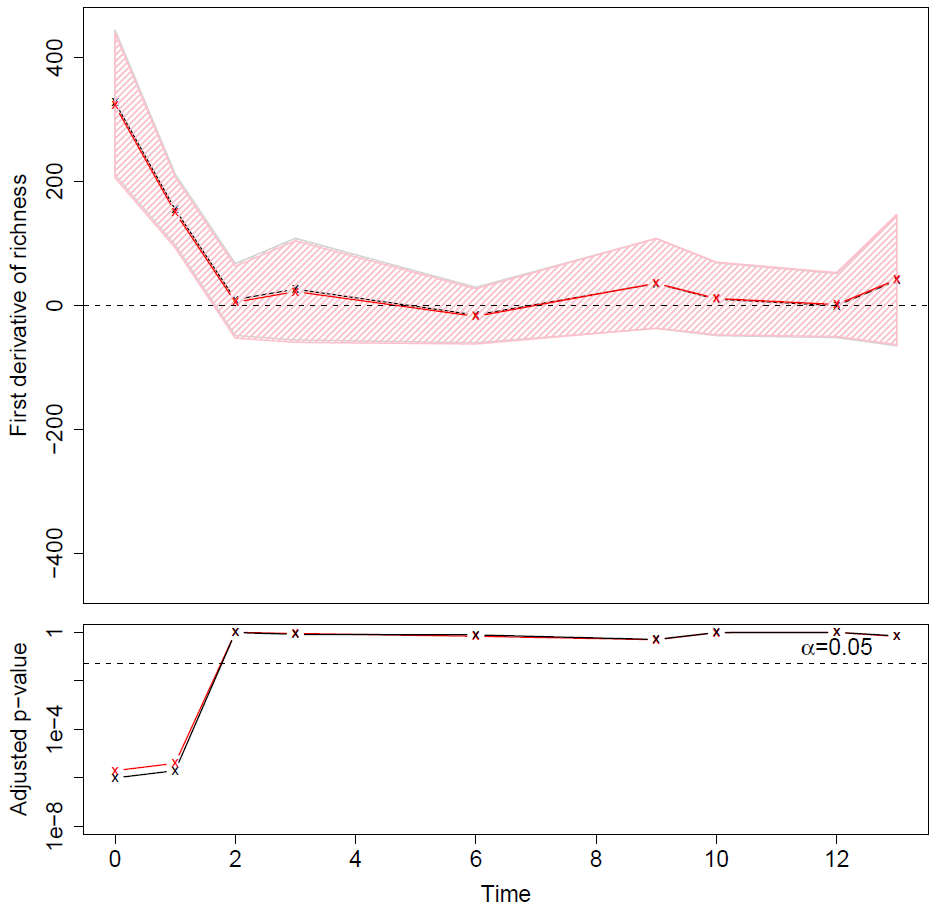


**Supplementary Figure 4B: First derivative of the richness of Fungi in the strawberry rhizosphere over 13 weeks of plant growth.** The top panel depicts the first derivative for peat and biochar-amended richness profiles in black and red, respectively. The shaded areas are simultaneous 95 % confidence bands that are estimated on a grid spanned by the observed time-points (t=0, 1, 2, 3, 6, 9, 10, 12, 13 weeks. The highest increase is observed at time zero and the increase in richness for both peat and biochar amended media moderates as plant growth progresses. For both profiles the first derivative is significantly higher than zero at week 0 and 1 at the 5% significance level. The bottom panel shows the corresponding p-value adjusted for multiple testing.


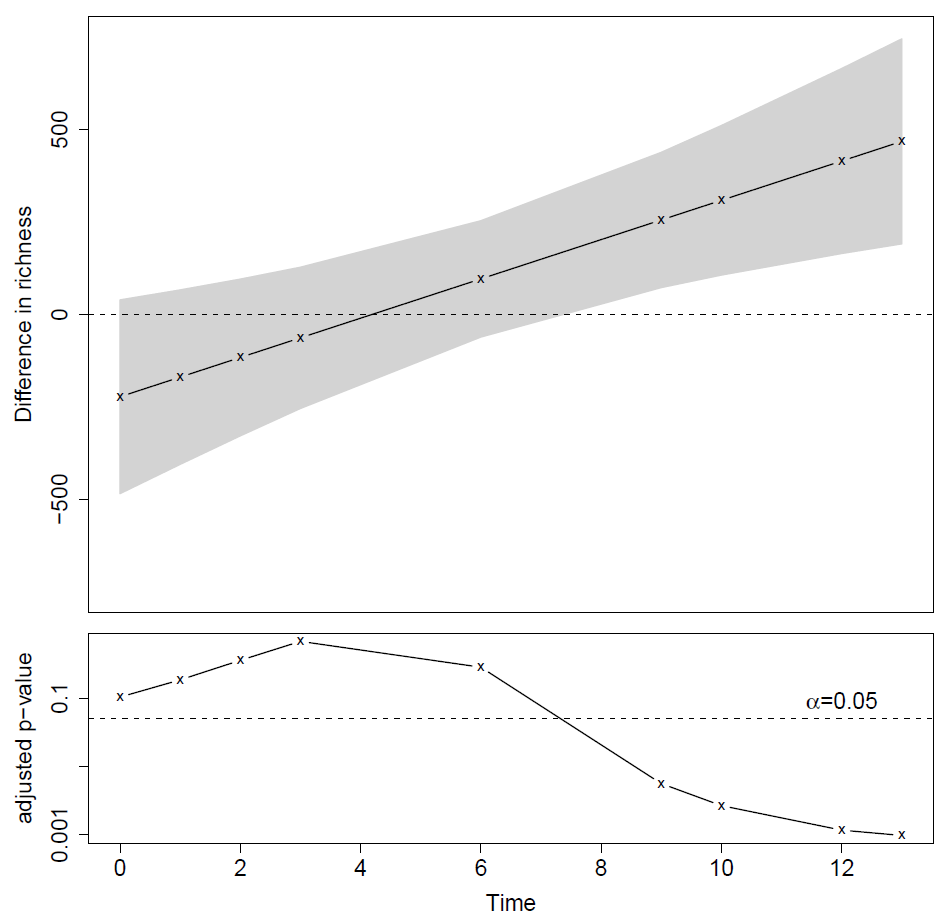


**Supplementary Figure 5A**: **Difference in bacterial richness of the strawberry rhizosphere over 13 weeks of plant growth in biochar amended and peat medium.** Top panel: the line represents the average difference in richness between biochar amended medium and peat medium. The shaded areas are simultaneous 95 % confidence bands that are estimated on a grid spanned by the observed time-points (t=0, 1, 2, 3, 6, 9, 10, 12, 13 weeks). Bottom panel: adjusted p-values corresponding to a two-sided test of the difference. The difference is not significant at the 5% significance level on week 0-6 and is significantly higher than 0 in week 9-13. Hence, from week nine onwards the bacterial richness in the rhizosphere of plants grown in biochar amended medium is on average significantly higher than that of plants grown in regular peat medium.


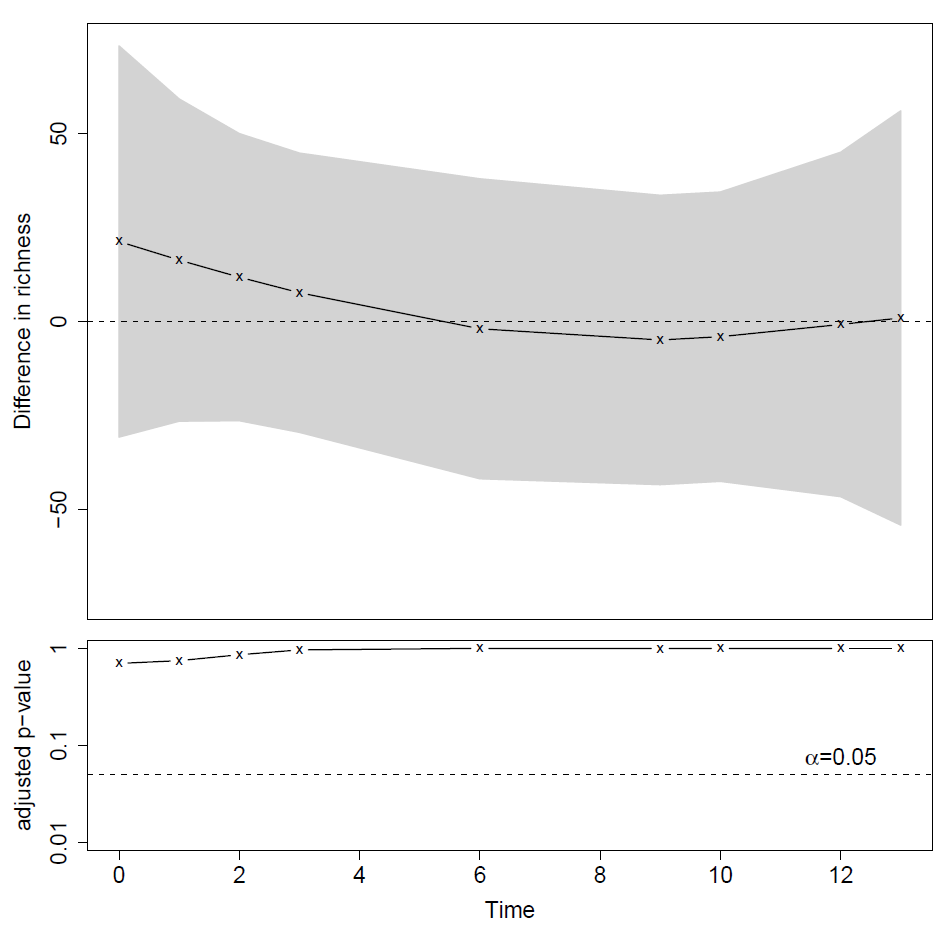


**Supplementary Figure 5B**: **Difference in fungal richness of the strawberry rhizosphere over 13 weeks of plant growth in biochar amended and peat medium.** Top panel: the line represents the average difference in richness between biochar amended medium and peat medium. The shaded areas are simultaneous 95 % confidence bands that are estimated on a grid spanned by the observed time-points (t=0, 1, 2, 3, 6, 9, 10, 12, 13 weeks). Bottom panel: adjusted p-values corresponding to a two-sided test of the difference. The difference is not significant at the 5% significance level on week 0-13.


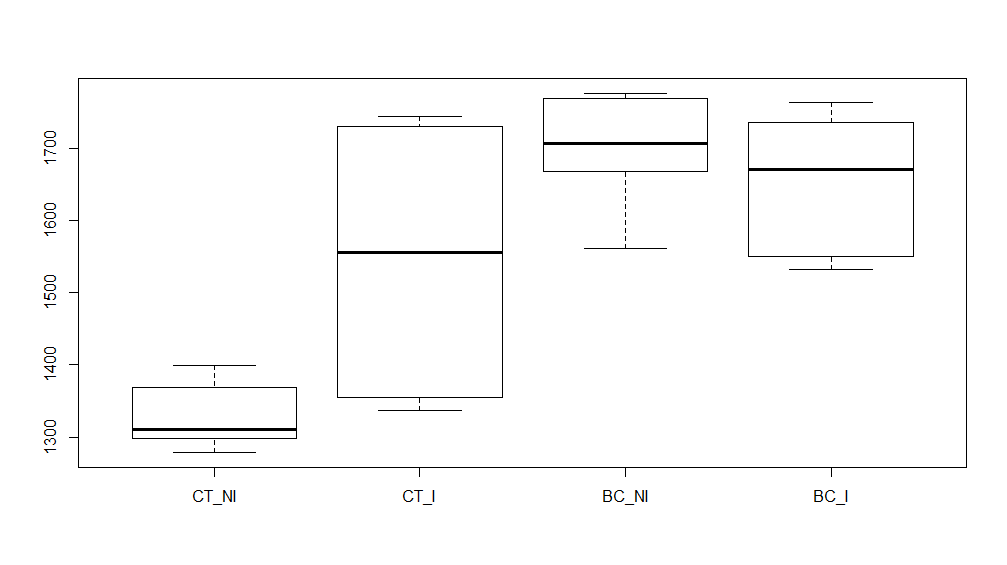


**Supplementary Figure 6**: Boxplots representing the richness (number of unique OTUs) of the bacteriome of the strawberry rhizosphere per treatment. CT= non-biochar treated peat, BC = biochar treated peat, NI = non-infected strawberry plants and I = B. cinerea infected strawberry plants.
